# Distinguishing nontuberculous mycobacterial lung disease and *Mycobacterium tuberculosis* lung disease on X-ray images using deep transfer learning

**DOI:** 10.1186/s12879-023-07996-5

**Published:** 2023-01-19

**Authors:** Minwoo Park, Youjin Lee, Sangil Kim, Young-Jin Kim, Shin Young Kim, Yeongsic Kim, Hyun-Min Kim

**Affiliations:** 1 Department of Laboratory Medicine, St. Vincent’s Hospital, The Catholic University of Korea, 93, Jungbu-Daero, Paldal-Gu, Suwon-Si, Gyeonggi-Do 16247 Republic of Korea; 2 Department of Mathematics, Pusan National University, 2, Busandaehak-Ro 63Beon-Gil, Geumjeong-Gu, Busan, 46241 Republic of Korea; 3 H.A.S. Inc., 24, Yeonje-Ro, Yeonje-Gu, Busan, 47605 Republic of Korea; 4 Department of Internal Medicine, St. Vincent’s Hospital, The Catholic University of Korea, 93, Jungbu-Daero, Paldal-Gu, Suwon-Si, Gyeonggi-Do 16247 Republic of Korea; 5 National Institute for Mathematical Sciences, 70, Yuseong-Daero 1689 Beon-Gil, Yuseong-Gu, Daejeon, 34047 Republic of Korea

**Keywords:** *Mycobacterium tuberculosis* lung disease, Nontuberculous mycobacterial lung disease, Deep learning, Chest X-ray image, Intersection over detected bounding-box

## Abstract

**Background:**

Nontuberculous mycobacterial lung disease (NTM-LD) and *Mycobacterium tuberculosis* lung disease (MTB-LD) have similar clinical characteristics. Therefore, NTM-LD is sometimes incorrectly diagnosed with MTB-LD and treated incorrectly. To solve these difficulties, we aimed to distinguish the two diseases in chest X-ray images using deep learning technology, which has been used in various fields recently.

**Methods:**

We retrospectively collected chest X-ray images from 3314 patients infected with *Mycobacterium tuberculosis* (MTB) or nontuberculosis mycobacterium (NTM). After selecting the data according to the diagnostic criteria, various experiments were conducted to create the optimal deep learning model. A performance comparison was performed with the radiologist. Additionally, the model performance was verified using newly collected MTB-LD and NTM-LD patient data.

**Results:**

Among the implemented deep learning models, the ensemble model combining EfficientNet B4 and ResNet 50 performed the best in the test data. Also, the ensemble model outperformed the radiologist on all evaluation metrics. In addition, the accuracy of the ensemble model was 0.85 for MTB-LD and 0.78 for NTM-LD on an additional validation dataset consisting of newly collected patients.

**Conclusions:**

In previous studies, it was known that it was difficult to distinguish between MTB-LD and NTM-LD in chest X-ray images, but we have successfully distinguished the two diseases using deep learning methods. This study has the potential to aid clinical decisions if the two diseases need to be differentiated.

**Supplementary Information:**

The online version contains supplementary material available at 10.1186/s12879-023-07996-5.

## Background

*Mycobacterium tuberculosis* (MTB) is a major causative organism of *Mycobacterium tuberculosis* lung disease (MTB-LD). It is one of the major causes of death from respiratory diseases, with 1.4 million deaths in 2019 [1]. Nontuberculous mycobacterial lung disease (NTM-LD), which has clinical symptoms similar to those of MTB-LD, is increasing worldwide in prevalence but is underestimated due to the impact of MTB-LD [2, 3]. NTM-LD is a lung disease caused by nontuberculous mycobacteria (NTM), which generally refers to mycobacteria other than MTB and *Mycobacterium leprae*. The increased prevalence of NTM-LD may be due to the increasing number of patients receiving immunosuppressive therapy for cancer or rheumatic disease [4].

Unfortunately, clinicians have difficulty differentiating MTB-LD from NTM-LD for the following reasons: First, acid-fast bacilli (AFB) staining is the standard test method for diagnosing MTB infection because it is fast and inexpensive [5]. However, it is known that MTB and NTM cannot be differentiated with AFB staining alone because they can be equally positive on AFB stain [6]. Second, because of these limitations of AFB staining, the gold standard to distinguish between MTB and NTM is a culture in liquid or solid media [7]. However, this has the disadvantage that it takes up to 8 weeks to differentially diagnose the two diseases [8]. Lastly, a polymerase chain reaction (PCR) test can be assumed to be tuberculosis if the result is positive, but it has the disadvantages of the risk of cross-contamination and that negative does not completely rule out the possibility of MTB-LD [9].

Because of these difficulties, NTM-LD is sometimes misdiagnosed as MTB-LD [10–12]. For example, follow-up research of patients diagnosed with MTB-LD found that 20% of patients were actually associated with NTM-LD rather than MTB-LD [13]. In addition, the research by Gomathy et al. reported that 39 out of 122 patients who did not respond to anti-tuberculosis treatment had NTM-LD other than MTB-LD [14]. Since an accurate diagnosis is essential for appropriate treatment, a misdiagnosis can lead to incorrect treatment, which can lead to various side effects of drugs and unnecessary medical expenses. In the worst case, it can lead to failure of treatment and adversely affect the patient's prognosis.

Previously, several studies have attempted to diagnose MTB-LD on chest X-ray or computed tomography (CT) radiographs using machine learning or deep learning [15, 16]. However, to our knowledge, no research has distinguished between MTB-LD and NTM-LD using deep learning on X-rays images. Some studies report difficulties in distinguishing between NTM-LD and MTB-LD using chest X-rays. [17, 18]. However, chest X-ray is still used first for diagnosing tuberculosis because of the advantage that it is faster and does not require a contrast agent compared to CT [19, 20].

Artificial intelligence technology has made remarkable achievements in areas known to be difficult in the medical field. Deep learning algorithms are widely used in radiology for tumor detection, segmentation, and disease prediction [21]. Driven by this trend, we aimed to distinguish between NTM-LD and MTB-LD in chest X-ray images by using various convolutional neural network (CNN) models and applying deep learning technologies, such as transfer learning. It can help patients with these diseases by supporting clinical decision-making by maximizing the benefits of chest X-ray examination.

## Methods

### Dataset

**Fig. 1:**
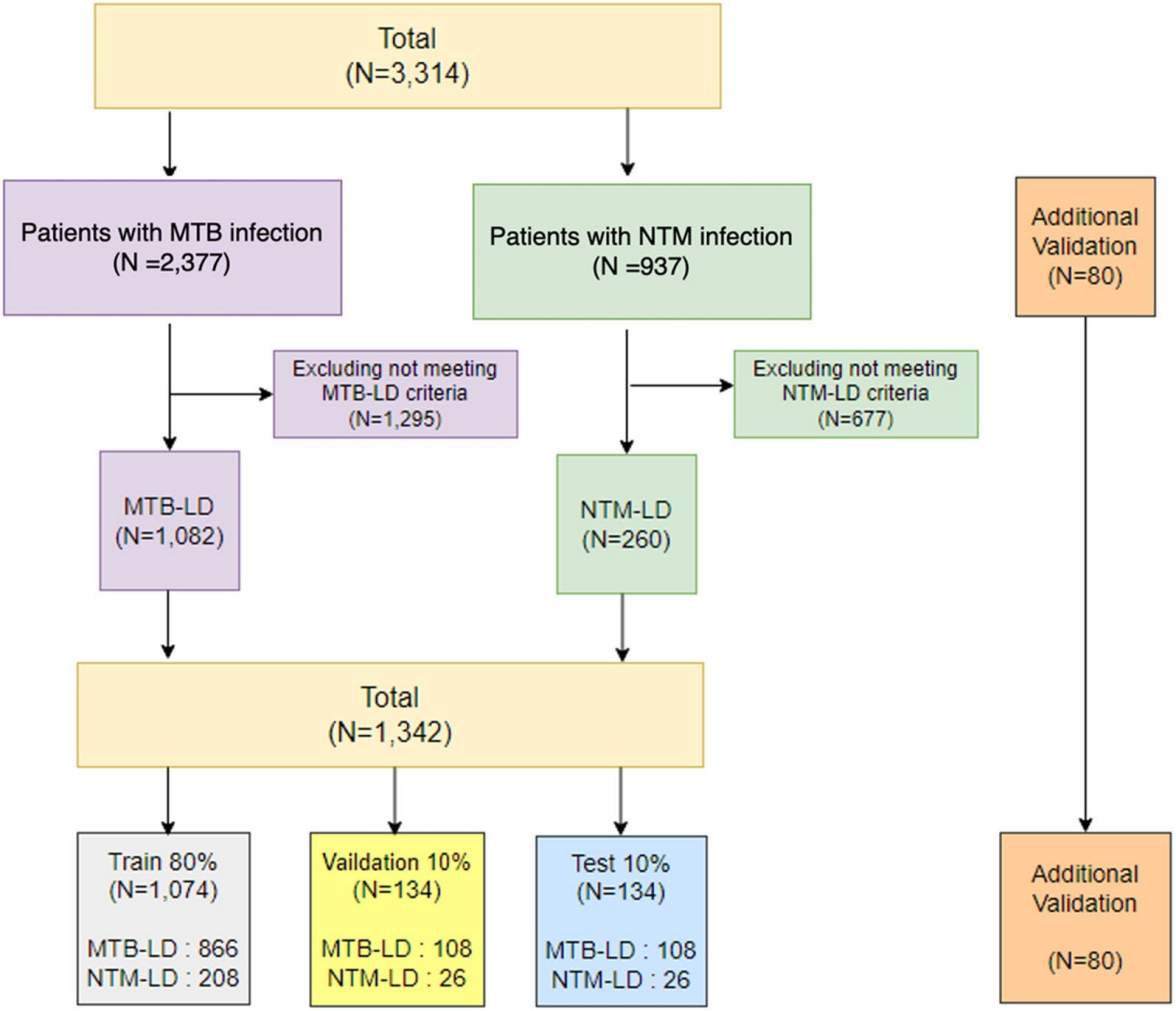
From the data selection, our final patients were chosen: MTB-LD (N = 1082), NTM-LD (N = 260), and additional validation (N = 80)

Chest X-ray images of 937 patients infected with NTM and 2377 patients infected with MTB were retrospectively collected at St. Vincent Hospital, the Catholic University of Korea, from January 1, 2010, to December 1, 2019. The collected patients were selected according to the diagnostic criteria shown in Table 1. Also, the data screening process can be found in Fig. 1. The diagnostic criteria were based on the Korean Guidelines for Tuberculosis, fourth edition [22], and the American Thoracic Society and Infectious Diseases Society of America (2007) [23]. After screening, 1082 MTB-LD patients and 260 NTM-LD patients were finally composed.

**Table 1:** Data selection criteria

MTB-LD	1. TB-PCR test positive
NTM-LD	1. Pulmonary or systemic symptoms
	– Cough, chest pain, dyspnea, fevers, hemoptysis
	– Other suspicious disease, such as tuberculosis, can be ruled out
	2. Radiologic
	– Nodular or cavitary opacities in chest radiograph
	3. Microbiologic
	– Positive culture results from at least two separate expectorated sputum or positive culture results from at least one bronchial wash or lavage

Considering the ratio between classes in the dataset, there was a data imbalance concerning the number of patients with MTB-LD and NTM-LD. Data imbalance has the potential to cause overfitting problems in deep learning. Most MTB-LD and NTM-LD patients have at least two images because chest X-rays are taken periodically for follow-up after diagnosis. In particular, NTM-LD has a very slow rate of radiological abnormal changes, and sufficient follow-up is required [24], so in our NTM-LD case, there were more chest X-ray images of one patient compared to MTB-LD. To resolve the data imbalance, chest X-ray images taken at the initial diagnosis were collected preferentially. And then, if there are two or more images from one patient, chest X-ray images taken on the date that the follow-up sputum culture test was positive were additionally collected. And the time restraint between collected chest X-ray and respiratory samples was limited to a maximum of one month. Because NTM-LD has a longer follow-up period and more sputum tests than MTB-LD, more NTM-LD images were collected per patient. This way, the proportion of patients was unbalanced, but chest X-ray images were as balanced as possible.

The entire data set was divided into three data sets for train, validation, and test at a ratio of 8:1:1, and we tried to make the average age and sex of patients in the three data set the same as possible. Also, by dividing by patient, not by image, we avoided distributing the same patients in the train, validation, and test datasets. In addition, multiple images were collected per patient, but for the test dataset, only one image per patient was used. For MTB-LD class in test data, a chest X-ray image taken on the day that the TB-PCR test was positive for the first time was selected. For NTM-LD class in test data, a chest X-ray image was selected when the secondary separation sputum test was positive. In summary, the final selected dataset in this study is shown in Table 2.

**Table 2:** The number of patients and chest X-ray images collected after screening

	MTB-LD	NTM-LD
Number of patients	1082	260
Number of chest X-ray images	2002	1462
Average number of images per patient	1.85	5.62

All collected data were approved by the IRB Ethics Committee of St. Vincent’s Hospital (IRB No. VC19WASI0305, VC22WASI0003). Due to the retrospective nature of the study and the use of fully anonymized clinical data, St. Vincent's Hospital's IRB Ethics Committee has waived the informed consent requirement.

### Image data preprocessing

Before deep learning training, chest X-ray images were pre-processed in two ways: Lung segmentation and Image augmentation. Lung segmentation, which extracts the lung region image from the original chest X-ray image, is widely used in the preprocessing stage of lung image analysis for clinical decision support systems (CDSS). Data augmentation refers to techniques that increase the size of training data to develop better deep learning models.

**Fig. 2:**
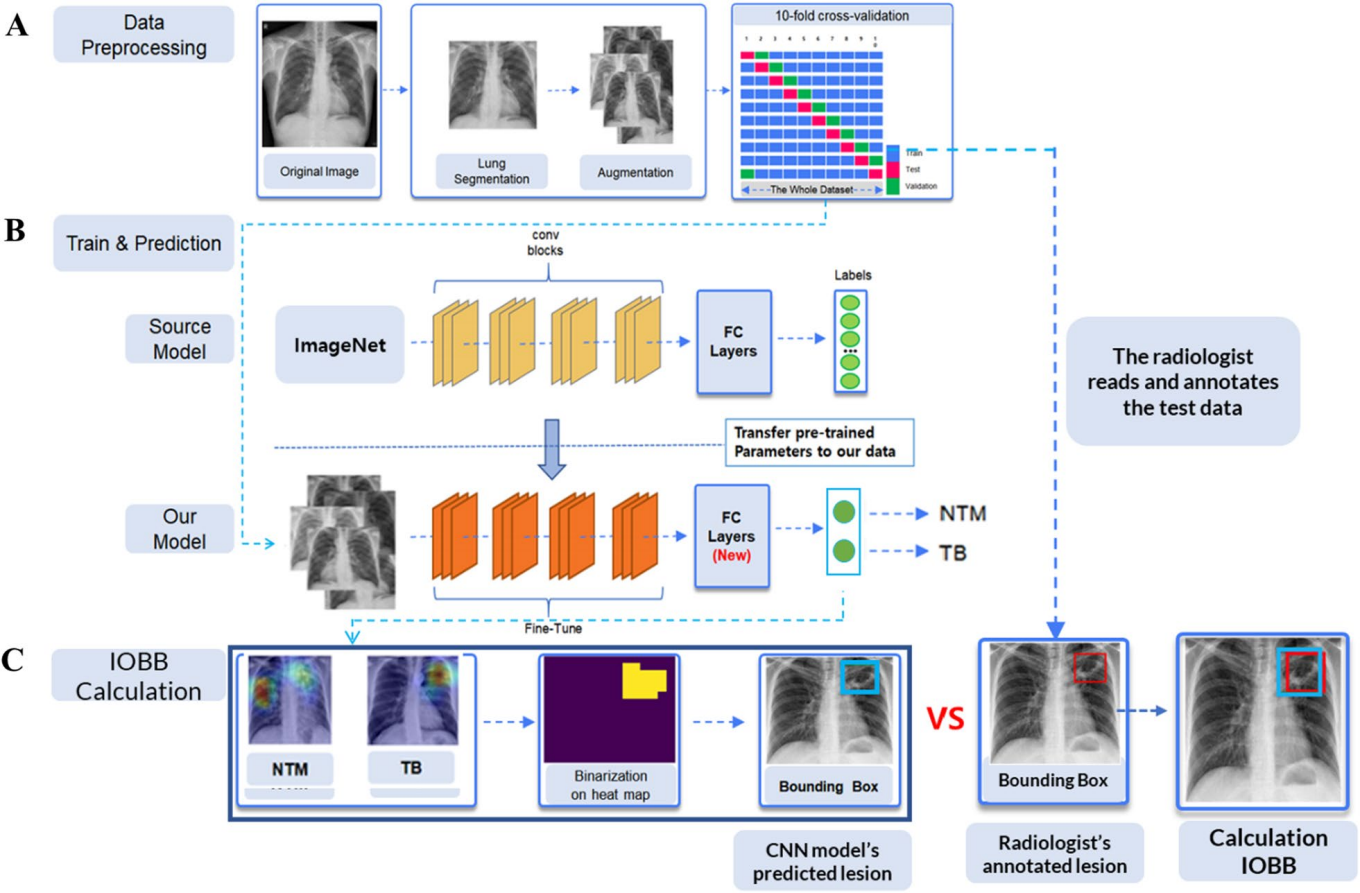
The entire process of our study

As shown in Fig. 2A, four edge coordinates (upper left, upper right, lower left, and lower right) of the lungs in the chest X-ray were computed using the open lung segmentation library [24]. Using the four coordinates calculated this way, the lung area was cut out from the original image and adjusted to 456 × 456 pixels. Afterward, image augmentation methods such as HorizontalFlip (flips the image horizontally around the y-axis) and ShiftScaleRotate (rotates the image left and right at a specified angle) were applied to the adjusted image. Augmented images were only used to train the deep learning model, and only the original images were used for validation and testing. This process was performed using an open augmentation library called Albumentations [25].

### Deep learning algorithms

Selecting an appropriate model for image diagnosis tasks using machine learning or deep learning is a key component [26]. First, three basic CNN models, such as Densenet 201 [27], ResNet 50 [28], and Efficientnet B4 [29] were used to create a model using train and validation data with transfer learning using ImageNet [30], and then evaluated on the test data. After that, three CNN models were compared using evaluation metrics such as precision, recall, F1 score, accuracy, and AUROC (area under the receiver operating characteristic). In all the models, a stochastic gradient descent was used as the optimizer with a momentum value of 0.9 and an initial learning rate of 0.001. In addition, we applied early stopping, learning rate adjustments, and drop-out to prevent overfitting. We applied a dropout rate of 0.1 just before the final activation function.

### Transfer learning

In general, learning CNN models requires a large amount of train data. However, in the medical field, it is often difficult to obtain the desired number of images. In these situations, transfer learning can be a good alternative. Transfer learning refers to the improvement of learning for a new task by transferring knowledge from a related task that has already been learned [31]. Transfer learning has demonstrated good performance in medical imaging applications, including training using chest X-ray and retinal fundus images, in the detection of Alzheimer’s disease, and the identification of skincare treatments [32]. With three CNNs, we applied transfer learning to boost the performance of the selected model via the fine-tuning method. We applied fine-tuning by removing the fully connected layers existing in DenseNet 201, ResNet 50, and EfficientNet B4 and replacing them with new fully connected layers, as shown in Fig. 2B.

### Ensemble method

Ensemble techniques help produce more accurate models by combining multiple trained models to reduce variance and bias [33]. There are many ways to apply ensemble techniques to deep learning models. In this study, a method of generating final output values from an ensemble in the form of averaging probability was used.

### Comparison of deep learning model and radiologist

We provided test data to a radiologist with ten years of experience to perform the readings to compare performance between models and the radiologist. In addition, we believe that it is more reasonable to predict NTM-LD and MTB-LD, if possible, based on abnormal lesions observed in these two lung diseases. To do so, the radiologist annotated the location of the lesion in each image in the form of a box while performing the reading for suspicious lung lesions comparison.

The overall comparative method and measurement metric for the lesion area was performed based on the study of Wang et al. [34], who studied abnormal lesions on chest X-rays. First, we compared the lesion annotated by the radiologist and the focused lesion of the model. To do so, we applied gradient-weighted class activation mapping (Grad-CAM) to predict which section of the lung image pertained to each specific diagnosis per model. Grad-CAM forms a map (referred to as a Grad-CAM map) that highlights the areas of the image that have a significant impact on the prediction based on gradient information that flows into the final convolutional layer [35]. By applying a 0.85 threshold value to heatmap vectors generated by Grad-CAM, we generated a bounding box that displays the lesion. The threshold implies that it converts values less than the threshold to 0 and values greater than the threshold to 1.

The selection of the threshold was based on prior research by Selvaraju et al. [35]. Bounding boxes were drawn along the boundaries for the largest pixel values among the pixels generated by setting the threshold. Figure 2C illustrates the whole process of visualization: Grad-CAM, binarization of the heatmap, and bounding box on the lung images. Bounding boxes generated by the model and the radiologist were compared using the IOBB metric, as shown in Fig. 2C. IOBB shows the extent of overlap between the two bounding boxes, and the IOBB value was calculated as

$$\mathrm{IOBB}= \frac{\mathrm{Area\,of\,Overlap}}{\mathrm{Area\,of\,Predicted\,Bounding}\text{-}\mathrm{Box\,by\,Model}}$$

A high IOBB value means that the box by our model is consistent with the anomalies determined by the radiologist.

### Additional validation

Despite collecting data for ten years to implement and evaluate deep learning models, we thought that there was a limit to accurately evaluating the performance of the model due to the lack of test data, especially for NTM-LD. In this case, external verification using public data or data from other institutions may be a good alternative. Unfortunately, to the best of our knowledge, there are currently no public data on NTM-LD. Also, it was not possible to obtain an external dataset due to our limited experimental conditions.

As an alternative, we collected data from new NTM-LD and MTB-LD cases that met the guidelines. From January 1, 2020, to December 31, 2021, chest X-ray images of 80 patients who met the diagnostic criteria were collected. The 80 patients consisted of 40 patients with NTM-LD and 40 with MTB-LD. These data are composed of completely different patients from the patient data used when implementing the deep learning models and newly generated data after implementing the deep learning model temporally. As with the test dataset, only one image was collected per patient, and the same data selection criteria were the same as those of the test dataset. We verified the performance of the deep learning models implemented using these newly collected data.

### Deep learning environment

The training was conducted in a computer environment with an Intel(R) Xeon(R) Gold 5120 CPU (2.20 GHz and 180 GB memory) and two V100 GPU cards under the Ubuntu 16.04 operating system. The CNN model was developed using TensorFlow with Keras, a deep learning framework for Python.

## Results

### Basic demographic and clinical information about the dataset

The final data set consisted of 1082 MTB-LD, 260 NTM-LD, and 80 additional validation, respectively. The demographic characteristics of the patients are shown in Table 3. In the independent t-test of the age of NTM-LD and MTB-LD patients, NTM-LD was significantly older than MTB-LD (p-value < 0.001). Also, in the chi-squared test for gender of NTM-LD and MTB-LD, there were significantly more males than females in MTB-LD (p-value < 0.001) but no statistically significant difference in NTM-LD (p-value = 0.2643). Additionally, information on age and gender for the train, validation, and test datasets for NTM-LD and MTB-LD can be found in Additional file 1: Table S1.

**Table 3:** Basic demographic and clinical information about the dataset

	MTB-LD	NTM-LD	Additional validation	
			MTB-LD	NTM-LD
Patients	1082	260	40	40
Average, age	58.0 ± 17.6	64.9 ± 16.1	57.6 ± 16.5	66.7 ± 15.3
Sex, male/female	653 (60.4%) /429 (39.6%)	121 (46.5%) /139 (53.5%)	23 (57.5%) / 17(42.5%)	18 (45.0%) / 22 (55.0%)
Cough	878 (81.1%)	205 (78.8%)	32 (80.0%)	30 (75.0%)
Chest pain	140 (12.9%)	29 (11.2%)	7 (17.5%)	6 (15.0%)
Dyspnea	10 (0.9%)	21 (8.1%)	3 (7.5%)	2 (5.0%)
Fever	379 (35.0%)	113 (43.5%)	17 (42.5%)	19 (47.5%)
Hemoptysis	178 (16.5%)	38 (14.6%)	8 (20.0%)	7 (17.5%)
Current smoking	140 (12.9%)	11 (4.2%)	5 (12.5%)	2 (5.0%)
Non-smoking	417 (38.5%)	151 (58.1%)	8 (20.0%)	10 (25.0%)
Smoked in the past	85 (7.9%)	15 (5.8%)	2 (5.0%)	1 (2.5%)
Unable to confirm smoking	440 (40.7%)	83 (31.9%)	25 (62.5%)	27 (67.5%)

In addition, Table 4 shows the information on nodular or cavity opacities on chest radiographs, which are radiographic characteristics of NTM-LD according to the guidelines and isolated NTM species through identification tests. Of the 260 patients diagnosed with NTM-LD, nodular opacities were found in 174 cases (66.9%), and cavitary opacities were found in 61 cases (23.5%) on chest X-rays. Both were found in 25 people (9.6%) *Mycobacterium avium complex* (MAC) accounted for most of the 203 (78.1%) strains of NTM identified through the identification test.

**Table 4:** Radiologic and microbiologic information of NTM-LD patients

Radiographic lesion type of NTM-LD patients, n (%) (N = 260)	
Nodular opacities	174 (66.9%)
Cavitary opacities	61 (23.5%)
Nodular and Cavitary opacities	25 (9.6%)

NTM Species, n (%) (N = 260)	
MAC (*M. avium-intracellulare* complex)	203 (78.1%)
*M. abscessus*	21 (8.1%)
*M. massiliense*	17 (6.5%)
*M. kansasii*	10 (3.8%)
*M. fortuitum*	3 (1.2%)
Others	6 (2.3%)

### Performance between different CNN models and comparison with the radiologist

**Fig. 3:**
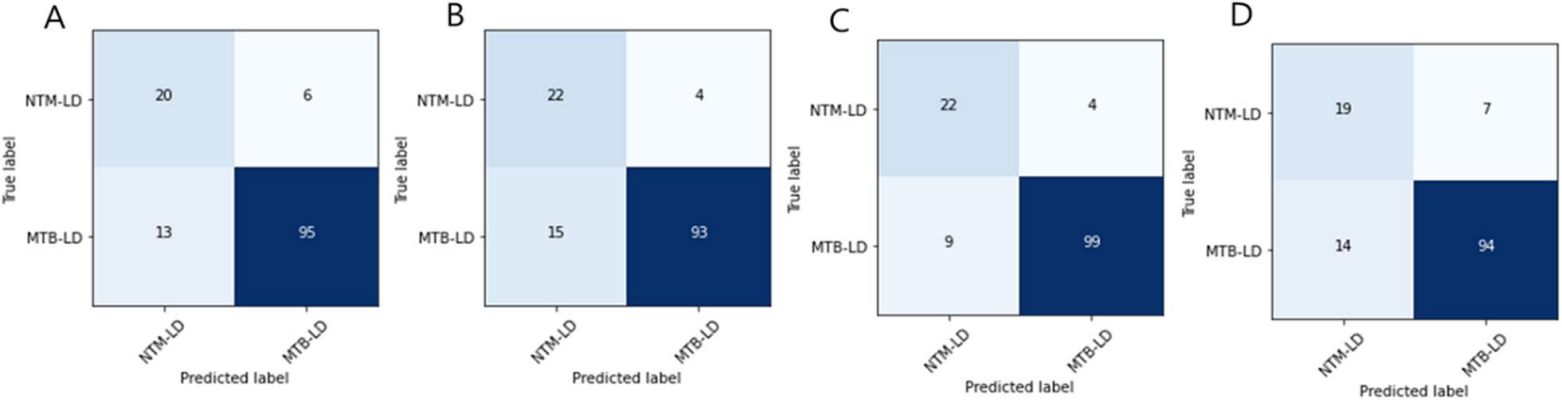
Confusion matrix of the DenseNet 201 (**A**), ResNet 50 (**B**), EfficientNet B4 (**C**), and the radiologist (**D**) on the test set

Three CNN models and radiologist results are shown in Table 5. Among the models, EfficientNet B4 performed the best in all evaluation metrics. The EfficientNet B4 model also showed the best performance in the quantitative evaluation of lung abnormalities using IOBB. An example figure of area comparison for the suspected lesion with the radiologist and the model using the IOBB can be found in Additional file 1: Fig. S1. In the performance comparison with the radiologist, most deep learning models showed higher results in the evaluation metrics. Figure 3 shows the confusion matrix for all CNN models and the radiologist on the test set. When Table 5 and Fig. 3 are comprehensively considered, it can be seen that the deep learning model is reasonably implemented.

**Table 5:** Three CNN models and the radiologist results for the test set

Class	Model	Precision	Recall	F1 score	AUROC	Accuracy	Average IOBB
NTM-LD (NP = 26, NI = 26)	DenseNet 201	0.61	0.77	0.68	0.82	0.77	0.24
	ResNet 50	0.59	**0.85**	0.70	0.85	**0.85**	0.27
	EfficientNet B4	**0.71**	**0.85**	**0.77**	**0.88**	**0.85**	**0.36**
	Radiologist	0.58	0.73	0.64	0.80	0.73	N/A
MTB-LD (NP = 108, NI = 108)	DenseNet 201	0.94	0.88	0.91	0.82	0.88	0.35
	ResNet 50	**0.96**	0.86	0.91	0.85	0.86	0.39
	EfficientNet B4	**0.96**	**0.92**	**0.94**	**0.88**	**0.92**	**0.50**
	Radiologist	0.93	0.87	0.90	0.80	0.87	N/A

Bold text means the highest performance, NP: Number of Patients, NI: Number of Images.

In the case of a radiologist in IOBB, it is used as ground truth and is marked as (N/A).

### Result of ensemble method

**Fig. 4:**
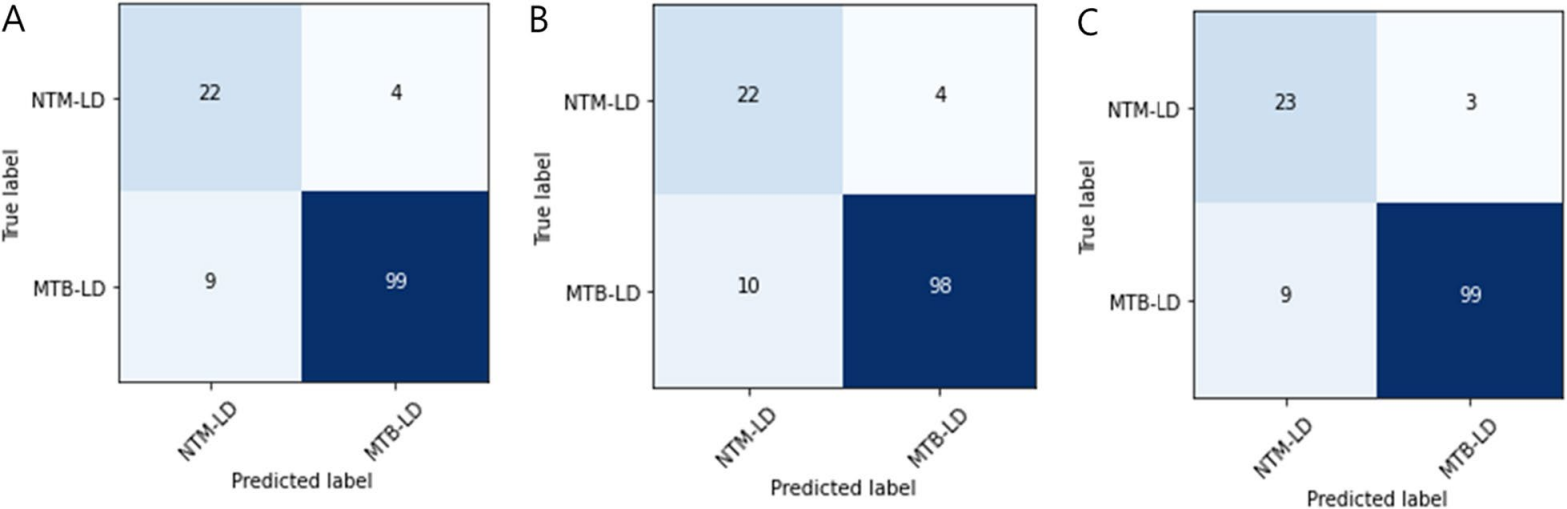
Confusion matrix of the EfficientNet B4 (**A**), Ensemble 1 (**B**), Ensemble 2 (**C**) on the test set

Table 6 and Fig. 4 show the results of Efficient B4, which showed the highest performance in individual models and two ensemble models in two combinations. Ensemble 1 is an ensemble of all three trained models, and Ensemble 2 is an ensemble of two models except DenseNet, which has the lowest performance among the three models. Ensemble 1 was not competitive compared to Efficient B4, but Ensemble 2 had a slight performance improvement over Efficient B4 overall.

**Table 6:** EfficientNet B4 and ensemble models results for the test set

Class	Model	Precision	Recall	F1 score	AUROC	Accuracy
NTM-LD (NP = 26, NI = 26)	EfficientNet B4	0.71	0.85	0.77	0.88	0.85
	Ensemble 1	0.69	0.85	0.76	0.88	0.85
	Ensemble 2	**0.72**	**0.88**	**0.79**	**0.90**	**0.88**
MTB-LD (NP = 108, NI = 108)	EfficientNet B4	0.96	**0.92**	**0.94**	0.88	**0.92**
	Ensemble 1	0.96	0.91	0.93	0.88	0.91
	Ensemble 2	**0.97**	**0.92**	**0.94**	**0.90**	**0.92**

Bold text means the highest performance, *NP* number of patients, *NI* number of images

Ensemble 1: DenseNet 201 + ResNet 50 + EfficientNet B4

Ensemble 2: ResNet 50 + EfficientNet B4

### Result of additional validation

**Fig. 5:**
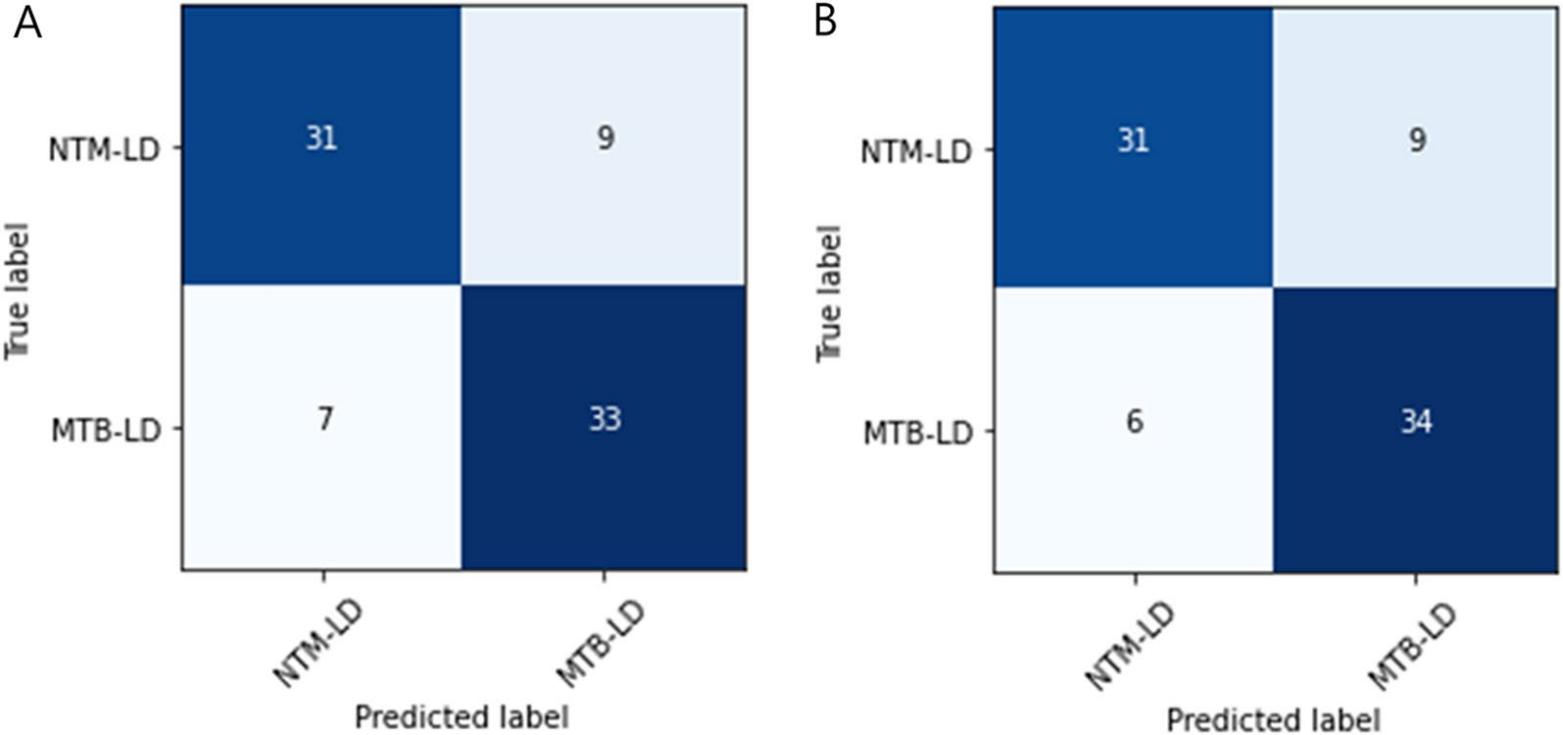
Confusion matrix of the EfficientNet B4 (**A**) and Ensemble 2 (**B**) on the additional validation dataset

Table 7 shows the results of predicting additional validation datasets using EfficientNet B4, which obtained the highest score among individual CNN models, and Ensemble 2 (ResNet 50 and EfficientNet B4), which obtained the highest score among ensemble models. Figure 5 also shows the confusion matrix of EfficientNet B4 and Ensemble 2. Ensemble 2 performed better than Efficient B4. In summary, the accuracy of both classes decreased on additional validation compared to the results on the test set. However, considering the imbalance between the two classes in the test set (NTM-LD: 26, MTB-LD: 108), NTM-LD performed better than the test set based on the F1 score. So, we thought that the model was reasonably implemented overall.

**Table 7:** Results of additional validation using the EfficientNet B4

Class	Model	Precision	Recall	F1 score	AUROC	Accuracy
NTM-LD (NP = 40, NI = 40)	EfficientNet B4	0.82	**0.78**	0.79	0.80	**0.78**
	Ensemble 2	**0.84**	**0.78**	**0.81**	**0.81**	**0.78**
MTB-LD (NP = 40, NI = 40)	EfficientNet B4	**0.79**	0.82	0.80	0.80	0.83
	Ensemble 2	**0.79**	**0.85**	**0.82**	**0.81**	**0.85**

Bold text means the highest performance, NP: Number of Patients, NI: Number of Images.

## Discussion

We aimed to distinguish between NTM-LD and MTB-LD using a clinically accessible and widely used chest X-ray image with a deep learning algorithm that has recently achieved remarkable results in the medical field. To do this, we conducted extensive experiments using various CNN models and implemented the optimal model. Additionally, we conducted the comparison with the radiologist and additional validation of newly diagnosed NTM-LD and MTB-LD patients. From the results, we concluded that the model was reasonably implemented.

There have been many studies previously to diagnose MTB-LD in chest X-rays using deep learning [36–38]. Still, no studies have distinguished NTM-LD from MTB-LD except for deep learning analysis study using CT images [18]. This may be due to the effect of some studies saying that it is impossible to distinguish NTM-LD from MTB-LD using chest X-rays [39]. To the best of our knowledge, our study is the first attempt to differentiate NTM-LD and MTB-LD in chest X-rays using deep learning. However, our deep learning model successfully predicted NTM-LD and MTB-LD using only chest X-ray images, demonstrating that chest X-rays can also help distinguish between the two diseases even before a CT scan.

This research had the following several limitations. First, there is the problem of imbalance between classes. Despite ten years of data collection, the number of NTM-LD patients (NTM-LD = 260) was one-third of the number of MTB-LD patients (MTB-LD = 1082). Therefore, we tried to solve this problem as much as possible by collecting more chest X-ray images every few months from one patient with NTM-LD compared to MTB-LD. This way, the number of patients was unbalanced, but the images were balanced as much as possible.

Second, validation using an external dataset was impossible due to our limited experimental conditions. To the best of our knowledge, there was no public dataset of NTM-LD patients, and no other hospital dataset could be utilized under our limited experimental conditions. To validate the deep learning model as much as possible within a given situation, we waited for a new NTM-LD and MTB-LD case that was completely different from the patients in the dataset used in the deep learning model. Finally, after a two-year data collection period, we collected 40 MTB-LD and 40 NTM-LD cases and used these data to validate the model successfully. Therefore, we have done our best for further validation within the given experimental conditions.

In summary, despite these limitations, we considered our study meaningful because it was the first to overcome the difficulty of distinguishing NTM-LD from MTB-LD in chest X-rays. It is challenging to apply the results of this study to clinical practice directly. However, if more data are obtained through future follow-up studies and a more robust deep learning model is created through verification, it can significantly help resolve the difficulty of distinguishing between the two diseases.

## Conclusions

Our research successfully distinguished NTM-LD and MTB-LD from lung disease patients with relatively high accuracy using a deep learning method. This research has the potential to be utilized as a tool for accurate diagnosis and appropriate treatment in situations where two diseases need to be distinguished.

## Supplementary Information

**Additional file 1: Table S1**. Demographic information about the train, validation, and test set. **Figure S1.** Example results. Top row: NTM-LD images with bounding boxes. Bottom row: MTB-LD images with bounding boxes. Examples of overlap between the bounding boxes generated by the radiologist (red) and by our model (blue) on lung images that were successfully predicted.

## Data Availability

The datasets supporting the conclusions of this article are included within the article. The raw image dataset in this research is not publicly available owing to the conditions of the ethics committee of St. Vincent’s Hospital to protect patient privacy. But the data may be released upon reasonable request to the corresponding author.

## Abbreviations

AFB: Acid-fast bacilli
CNN: Convolutional neural network
CT: Computed tomography
Grad-CAM: Gradient-weighted class activation mapping
IOBB: Interaction over detected B-Box
MTB-LD: *Mycobacterium tuberculosis* Lung disease
NTM-LD: Nontuberculous mycobacterial lung disease
PCR: Polymerase chain reaction
AUROC: Area under the receiver operating characteristic
