# Supplementary material for: Distinguishing nontuberculous mycobacterial lung disease and Mycobacterium tuberculosis lung disease on X-ray images using deep transfer learning
Source: BMC Infect Dis. 2023 Jan 19;23:32. doi: 10.1186/s12879-023-07996-5 (PMC9854086; doi:10.1186/s12879-023-07996-5)
Supplement: Supplementary file 1 — Additional file 1: Table S1. Demographic information about the train, validation, and test set. Figure S1. Example results. Top row: NTM-LD images with bounding boxes. Bottom row: MTB-LD images with bounding boxes. Examples of overlap between the bounding boxes generated by the radiologist (red) and by our model (blue) on lung images that were successfully predicted. [file 12879_2023_7996_MOESM1_ESM.docx]

**Distinguishing nontuberculous mycobacterial lung disease and Mycobacterium tuberculosis lung disease on X-ray images using deep transfer learning**

Minwoo Park^1^, Youjin Lee^2^*, Sangil Kim^2^*, Young-Jin Kim^3^, Shin Young Kim^4^, Yeongsic Kim^1^, Hyun-Min Kim^5^

^1^Department of Laboratory Medicine, St. Vincent’s Hospital, The Catholic University of Korea, 93, Jungbu-daero, Paldal-gu, Suwon-si, Gyeonggi-do 16247, Republic of Korea

^2^Department of Mathematics, Pusan National University, 2, Busandaehak-ro 63beon-gil, Geumjeong-gu, Busan 46241, Republic of Korea

^3^H.A.S. Inc., 68, Busandaehak-ro 50beon-gil, Geumjeong-gu, Busan 46285, Republic of Korea

^4^Department of Internal Medicine, St. Vincent’s Hospital, The Catholic University of Korea, 93, Jungbu-daero, Paldal-gu, Suwon-si, Gyeonggi-do 16247, Republic of Korea

^5^National Institute for Mathematical Sciences, 70, Yuseong-daero 1689beon-gil, Yuseong-gu, Daejeon 34047, Republic of Korea

***Corresponding Authors**: Youjin Lee and Sangil Kim

Department of Mathematics, Pusan National University

2, Busandaehak-ro 63beon-gil, Geumjeong-gu, Busan 46241, Republic of Korea

Phone: (+82) 051-510-2209

Fax: (+82) 051-581-1458

Email: youjin.lee@pusan.ac.kr (YL); sangil.kim@pusan.ac.kr (SK)

**Additional Information**

**Table S1**. Demographic information about the train, validation, and test set.

|  | NTM-LD (N=260) | | | MTB-LD (N=1082) | | |
| --- | --- | --- | --- | --- | --- | --- |
|  | Train  (n = 208) | Validation  (n = 26) | Test  (n = 26) | Train  (n = 866) | Validation  (n = 108) | Test  (n = 108) |
| Age | 66.4  ±16.2 | 64.8  ±16.1 | 65.5  ±14.9 | 58.3  ±16.9 | 60.9  ±18.9 | 59.1  ±14.4 |
| Sex,  male/  female | 98  (47%)  /110  (53%) | 11  (42%)  /15  (58%) | 12  (46%)  /14  (54%) | 516  (60%)  /350  (40%) | 66  (61%)  /42  (39%) | 71  (66%)  /37  (37%) |


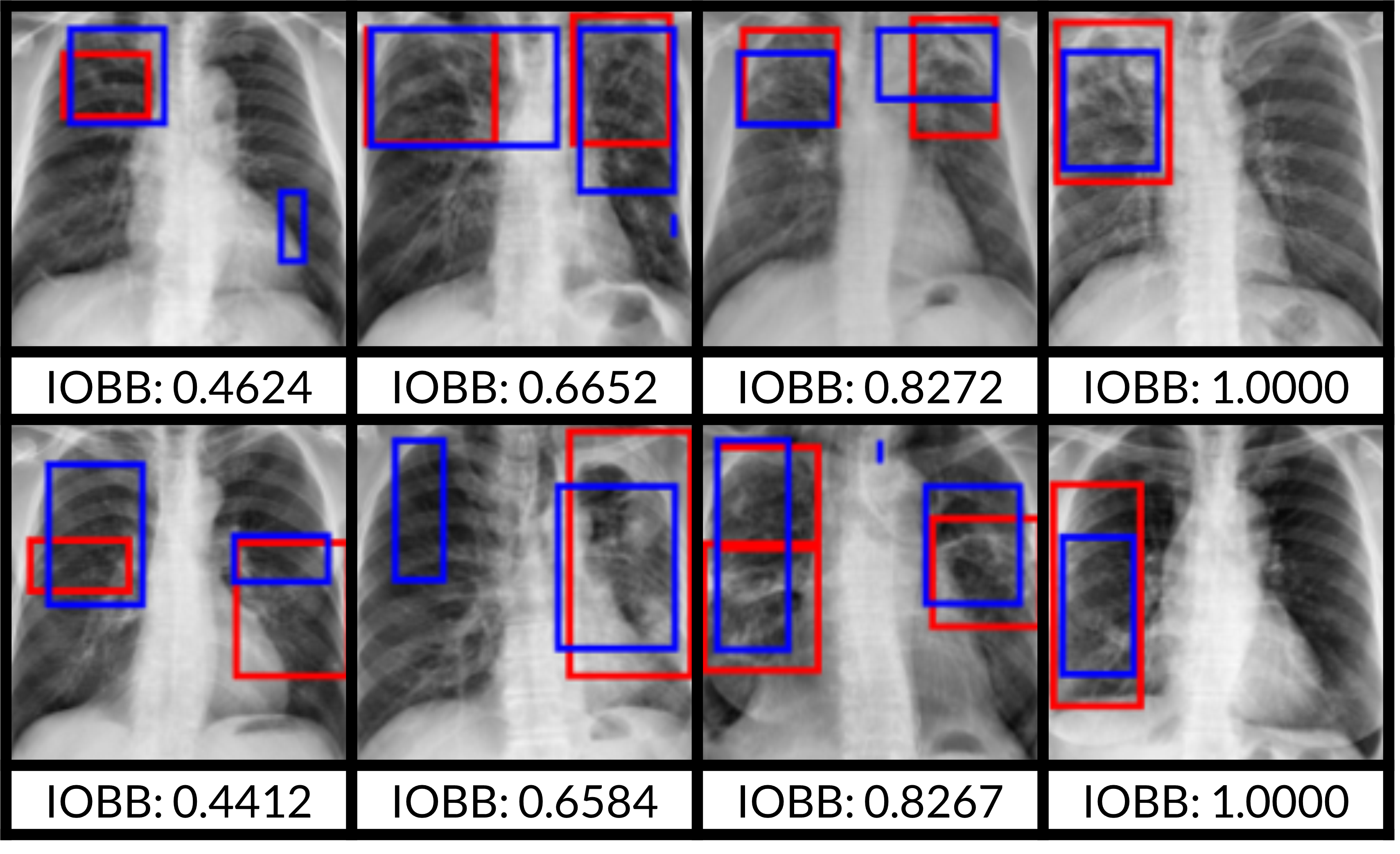


**Figure S1.** Example results. Top row: NTM-LD images with bounding boxes. Bottom row: MTB-LD images with bounding boxes. Examples of overlap between the bounding boxes generated by the radiologist (red) and by our model (blue) on lung images that were successfully predicted.
